# LRRC8A: A multifaceted regulator in cancer, neurological disorders, metabolic diseases and immune modulation

**DOI:** 10.1016/j.gendis.2025.101773

**Published:** 2025-07-16

**Authors:** Longjun Yang, Qiang Ding, Xiaoyu Ji, Panpan Lu, Mei Liu

**Affiliations:** Tongji Hospital of Tongji Medical College of Huazhong University of Science and Technology, Department of Gastroenterology, Wuhan, Hubei 430000, China

**Keywords:** Immunity, LRRC8A, Molecular mechanism, Structure, VRAC

## Abstract

Leucine-rich repeat containing 8A (LRRC8A) is a member of the LRRC8 family, exhibiting broad expression across various tissues and cells in vertebrates. Like other LRRC8 family members, LRRC8A contributes to the formation of volume-regulated anion channels (VRACs), which are crucial for regulating cell volume and maintaining homeostasis. LRRC8A participates in diverse signaling pathways. Multiple studies have validated the links between LRRC8A dysregulation and neurological disorders, metabolic ailments, and tumors. This review provides a comprehensive overview of the regulatory mechanisms of LRRC8A in these pathologies. The primary goal was to assess the potential of LRRC8A as a therapeutic target for treating diseases and address key unresolved issues.

## Introduction

Leucine-rich repeat-containing protein 8A (LRRC8A), also known as volume-regulated anion channel regulatory factor 1 (VRACR1), is a core component of volume-regulated anion channels (VRACs).^1^^,^^2^ In instances of cellular swelling due to osmotic changes, VRACs play a vital role in regulating cellular volume by promoting the efflux of chloride ions and small organic osmolytes, leading to a reduction in cell volume.^3^^,^^4^ The LRRC8 family comprises five members: LRRC8A, LRRC8B, LRRC8C, LRRC8D, and LRRC8E.^5^^,^^6^ Among them, LRRC8A is fundamental, as it is indispensable for VRAC assembly and optimal function.^1^ Other LRRC8 proteins heteromerically coassemble with LRRC8A to create VRACs with unique properties and functions.^7^, ^8^, ^9^ In addition to its role in osmoregulation, LRRC8A is involved in diverse other cellular processes, including lipid synthesis, insulin signaling, apoptosis, and cell proliferation.^10^, ^11^, ^12^, ^13^ The diverse functions of LRRC8A indicate its potential contribution to pathologies. Furthermore, recent research has demonstrated the involvement of LRRC8A in cancer progression, with several studies indicating its overexpression in various cancers and its correlation with drug resistance.^14^

While the significance of LRRC8A has been growing, our grasp of its involvement and regulatory pathways in diseases is still at a nascent stage. Recent advancements have considerably enhanced our comprehension of the structural foundations of VRAC formation and operation, the linkage between LRRC8A dysregulation and illnesses, as well as the precise regulatory mechanism. A comprehensive investigation into the structure–function relationship, regulatory mechanisms, and disease associations of LRRC8A advances our understanding of fundamental biological processes. This finding opens new avenues for developing targeted therapeutics and treatment strategies directed at VRAC channels. This review aims to provide a comprehensive overview of current insights into LRRC8A, examining its diverse roles in health and disease and evaluating its potential as a therapeutic target. In the era of precision medicine, LRRC8A research represents the frontier of translational medicine, bridging molecular channel studies with clinical applications and offering potential breakthroughs for diagnosing and treating challenging diseases.

## Structure of LRRC8A

**Figure 1:**
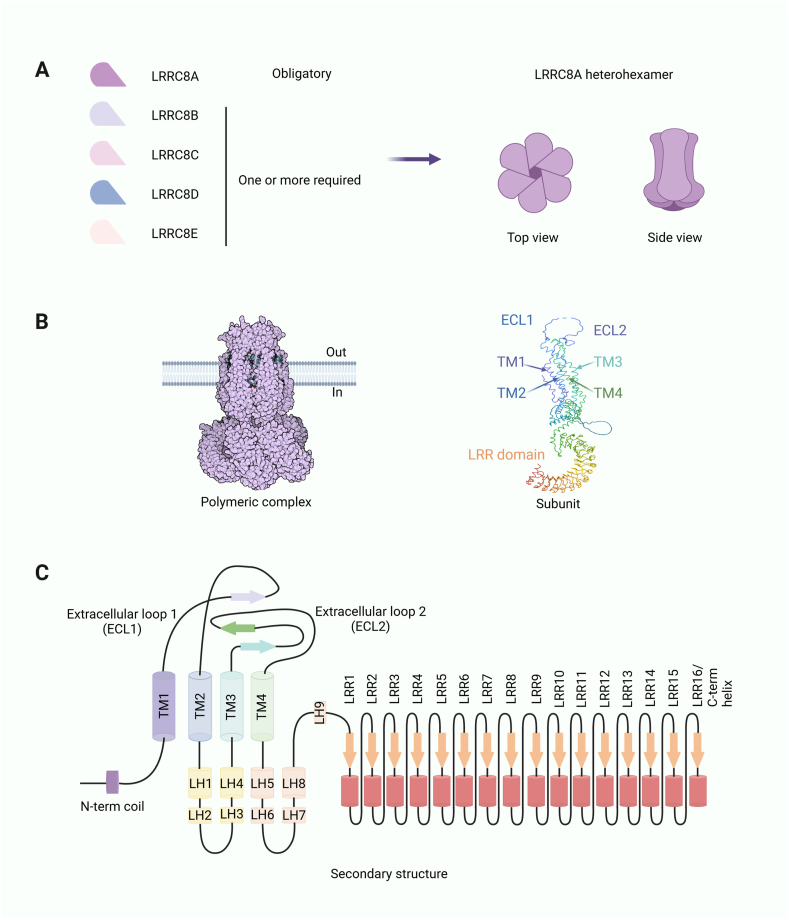
Structures of the LRRC8 heterohexamer and monomer. **(A)** A planar schematic diagram of LRRC8A/VRAC. **(B)** 3D structure diagram of LRRC8A/VRAC. **(C)** Secondary structure diagram of LRRC8A. ECL, extracellular loop; TM, transmembrane; LH, leucine-rich repeat helix; LRR, leucine-rich repeat.

LRRC8A is a transmembrane protein belonging to the LRRC8 family that plays a vital role in maintaining ion balance inside and outside the cell and responding to cellular stress.^15^^,^^16^ LRRC8A consists of four parts: an N-terminal intracellular region, a transmembrane region, an extracellular ring region, and a C-terminal intracellular region.^17^ The N-terminal intracellular region and the C-terminal intracellular region are primarily responsible for regulating channel function. The transmembrane region comprises four α-helices, forming an ion-conducting channel, whereas the extracellular ring region consists of seventeen leucine-rich repeat sequences, resulting in a horseshoe-shaped structure.^18^, ^19^, ^20^, ^21^ The primary role of LRRC8A is to form the VRACs. LRRC8A combines with the subunits of other LRRC8 family members to create a hexameric structure, which serves as the fundamental unit of the VRAC channel (Fig. 1).^7^ VRAC serves as a multifunctional anion channel, facilitating the release of chloride ions and organic osmolytes like taurine and inositol phosphate,^22^, ^23^, ^24^, ^25^ thereby regulating ion distribution across the membrane and maintaining cell volume. Additionally, VRACs play a role in regulating various cellular functions by transmitting signaling molecules involved in processes like the cell cycle, apoptosis, and cell migration.^3^^,^^26^^,^^27^ Within the LRRC8 family, LRRC8A combines with other members to form heteromeric VRAC channels with distinct properties. For example, the presence of LRRC8D enhances channel selectivity for taurine and certain chemotherapeutic agents,^8^ while LRRC8C increases amino acid selectivity.^28^ In summary, LRRC8A is the central component of the VRAC channel and is essential for maintaining ion balance, responding to cellular stress, and regulating cell behavior across numerous physiological and pathological contexts (Fig. 1).

## The role of LRRC8A in human diseases

LRRC8A is involved in various physiological processes and has been confirmed to be associated with conditions such as cancer, neurological disorders, and metabolic diseases. A deep understanding of the role and specific molecular mechanisms of LRRC8A in diseases is instructive for targeted precision therapy.

## LRRC8A in cancer

### Cancer development and progression

Numerous studies have established that LRRC8A contributes to the progression of multiple malignancies, facilitating tumor development and growth through various signaling pathways and cellular mechanisms (Table 1). In esophageal squamous cell carcinoma, LRRC8A promotes uncontrolled cell proliferation and invasive metastasis by relieving G1/S phase checkpoint control through the suppression of the cyclin-dependent kinase (CDK) inhibitors p21 and p27, as well as the inhibition of transcription repressor E2F7 activity; simultaneously, it up-regulates the matrix metalloproteinase MMP1 to enhance extracellular matrix degradation and increases integrin ITGAvα expression to alter cell–matrix interactions.^29^ These coordinated transcriptional changes suggest that LRRC8A functions as a master regulator of malignant transformation in esophageal cancer, representing a promising therapeutic target capable of simultaneously disrupting multiple oncogenic pathways. In gastric cancer, LRRC8A maintains intracellular chloride concentrations to support cancer cell survival under osmotic stress conditions. Additionally, it disrupts p53 protein stability to block apoptotic signal transduction.^30^ In light of these findings, targeting LRRC8A can simultaneously address metabolic adaptation and apoptosis resistance issues in gastric cancer.^30^ In hepatocellular carcinoma (HCC), LRRC8A activates the JNK MAPK cascade, leading to the phosphorylation and activation of the c-Jun transcription factor. This activation induces the expression of Snail and Twist, which transcriptionally repress E-cadherin while increasing vimentin and N-cadherin expression, thereby promoting epithelial–mesenchymal transition (EMT) of cancer cells.^31^ This study indicates that targeting the JNK pathway or disrupting LRRC8A channel function may prevent hepatocellular carcinoma metastasis. In colorectal cancer, LRRC8A interacts with PIP5K1B to promote PIP2 (phosphatidylinositol^4^^,^^5^-bisphosphate) production. PIP2 functions either directly as a second messenger or is converted to others, such as inositol 1,4,5-trisphosphate (IP3), diacylglycerol (DAG), or phosphatidylinositol^3^, ^4^, ^5^-trisphosphate (PIP3), which participate in cell signaling pathways that drive tumor progression and metastasis.^32^^,^^33^ Moreover, the up-regulation of LRRC8A can increase the formation and secretion of extracellular vesicles (EVs) in colorectal cancer cells, and LRRC8A is concomitantly excreted along with the extracellular vesicle contents into the extracellular environment, where extracellular vesicle-bound LRRC8A acts as a regulator to preserve the stability of the extracellular vesicle volume in response to alterations in extracellular osmotic pressure.^34^ This phenomenon suggests that LRRC8A may promote intercellular communication within the tumor microenvironment, potentially facilitating cancer cell survival, immune evasion, and the preparation of metastatic niches. Future therapeutic strategies for colorectal cancer might focus on disrupting the LRRC8A-PIP5K1B interaction or blocking LRRC8A incorporation into EVs using specific blocking antibodies or small molecule inhibitors. In cervical cancer, the RNA methyltransferase NOL1/NOP2/Sun domain family member 2 (NSUN2) stabilizes LRRC8A mRNA through m5C modification, activating the PI3K-AKT signaling pathway, which ultimately suppresses cell apoptosis and facilitates tumorigenesis.^35^ This research reveals several potential therapeutic targets, including NSUN2 inhibitors, to decrease LRRC8A mRNA stability or direct LRRC8A antagonists to block downstream PI3K-AKT activation. Research on pancreatic cancer has revealed that the influence of LRRC8A extends beyond its intrinsic effects on cancer cells to the tumor microenvironment. High LRRC8A expression is associated with altered immune cell infiltration patterns, particularly decreased CD8^+^ T cells and increased myeloid-derived suppressor cells (MDSCs).^36^ Although the specific regulatory mechanisms remain unclear, this study suggests that LRRC8A contributes to immune evasion and creates a pro-tumor microenvironment, explaining why high LRRC8A expression serves as a negative prognostic indicator for pancreatic cancer patients. LRRC8A-targeted therapeutic strategies for pancreatic cancer may not only directly affect cancer cells but also help reverse immunosuppression, enhancing the efficacy of immunotherapies.

**Table 1:** The oncogenic roles of LRRC8A in different types of cancer.

Cancer type	Function	Reference
**Colon cancer**	Promotes proliferation, metastasis, and drug resistance; volume regulation.	32, 33, 34,40
**Gastric cancer**	Promotes proliferation.	30
**Glioblastoma**	Promotes proliferation, inhibits the responsiveness to temozolomide and carmustine.	39,43
**Cervical cancer**	Promotes proliferation and metastasis.	35
**Ovarian cancer**	Maintains VRAC activity; promotes intracellular accumulation of cisplatin; facilitates cisplatin-induced apoptosis.	14,44
**Glioma**	Promotes temozolomide-induced apoptosis.	37
**Pancreatic adenocarcinoma**	Promotes metastasis; promotes CD8^+^ T cells, neutrophils, and dendritic cells infiltration.	36
**Alveolar carcinoma**	Facilitates cisplatin-induced apoptosis.	38
**Esophageal squamous cell carcinoma**	Promotes proliferation and metastasis.	29
**Oral squamous cell carcinoma cells**	Maintains VRAC activity; promotes proliferation.	13
**Breast cancer**	Promote migration and invasion.	28,45
**Liver cancer**	Promotes G1/S transition and EMT.	31

The therapeutic significance of targeting LRRC8A varies across different cancer types but shares common potential. For esophageal and gastric cancers, LRRC8A inhibition may restore cell cycle checkpoints and reactivate p53-mediated tumor suppression. In hepatocellular carcinoma, disrupting the LRRC8A-JNK axis could prevent metastasis. For colorectal cancer, targeting the LRRC8A-PIP5K1B interaction may simultaneously inhibit multiple oncogenic signaling cascades. In cervical cancer, destabilizing LRRC8A mRNA through NSUN2 inhibition or directly antagonizing LRRC8A function could reactivate apoptotic pathways. For pancreatic cancer, LRRC8A inhibition presents the intriguing possibility of converting an immunosuppressive microenvironment to an immunopermissive one, potentially synergizing with checkpoint inhibitor immunotherapies.

### LRRC8A and drug resistance

Drug resistance is a significant contributor to treatment failure in cancer. Exploring the mechanisms underlying drug resistance is vital for developing strategies to overcome resistance and enhance treatment efficacy. While numerous studies have linked LRRC8A to drug resistance, its role varies across different cancer types. For instance, the overexpression of LRRC8A boosts temozolomide-induced apoptosis in U87/R glioma cells by facilitating the mitochondrial apoptotic pathway.^37^ Decreased LRRC8A expression reduces platinum drug uptake in p53-deficient breast cancer and ovarian cancer cells^14^^,^^28^ and decreases the expression and activation of p53, MDM2 (mouse double minute 2 homolog), and p21 in platinum-treated A549 lung adenocarcinoma cells, resulting in reduced platinum-induced apoptosis.^38^ In contrast, silencing LRRC8A enhances the responsiveness of glioblastoma to temozolomide and carmustine.^39^ Additionally, LRRC8A has been implicated in developing oxaliplatin resistance in colon cancer cells.^40^ These contentious conclusions could be attributed to the following factors. First, LRRC8A necessarily assembles with other LRRC8 family members to constitute functional heterohexameric ion channels. Although LRRC8A represents an essential component of all functional VRACs, the specific identity of its companion subunits critically determines drug selectivity. VRAC complexes containing the LRRC8D subunit, for example, mediate approximately 50% of cisplatin uptake under isotonic conditions, while complexes incorporating LRRC8C or LRRC8E subunits contribute negligibly to cisplatin transport.^8^ This molecular specificity elucidates why assessments focused exclusively on LRRC8A expression levels, without consideration of its companion subunits, frequently yield contradictory clinical observations. Consequently, in evaluating VRAC-mediated drug transport, the integrated expression profile of LRRC8A alongside its specific companion subunits should be analyzed as a functional unit to determine their collective impact on the therapeutic response accurately. Second, the tumor microenvironment, particularly the extracellular osmolarity and pH, directly influences VRAC activation patterns,^41^^,^^42^ which may affect the role of LRRC8A in drug resistance. Moreover, LRRC8A interacts with multiple signaling pathways, which are activated to varying degrees across different cancer types, resulting in context-dependent outcomes for chemotherapeutic sensitivity.

**Figure 2:**
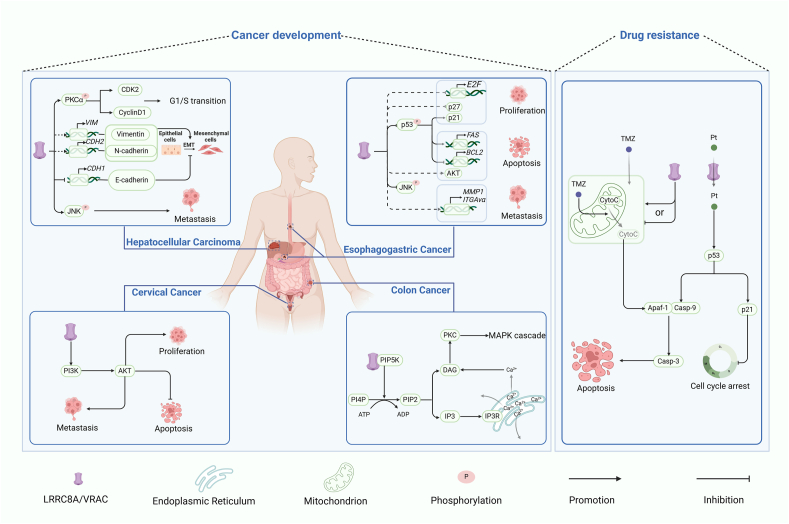
LRRC8A promotes cancer progression and influences drug resistance.

In conclusion, personalized strategies can be employed for tumors with varying LRRC8A expression patterns. For platinum-resistant tumors exhibiting low LRRC8A expression, combination therapy with VRAC activators may increase drug uptake, whereas VRAC inhibitors can function as chemosensitizers in cancers where LRRC8A promotes resistance. More precise strategies include developing selective compounds targeting specific LRRC8 isoforms (particularly LRRC8A-LRRC8D heteromers) to optimize drug uptake while minimizing non-specific effects. Furthermore, LRRC8A expression patterns and isoform composition may serve as predictive biomarkers to guide individualized treatment decisions. Future investigations should utilize patient-derived xenografts and organoid models to elucidate the mechanisms by which LRRC8A contributes to drug resistance across diverse genetic backgrounds, thereby establishing a foundation for novel therapeutic interventions and improved chemotherapeutic efficacy, ultimately enhancing clinical outcomes for cancer patients (Fig. 2).

### LRRC8A in neurological disorders

**Figure 3:**
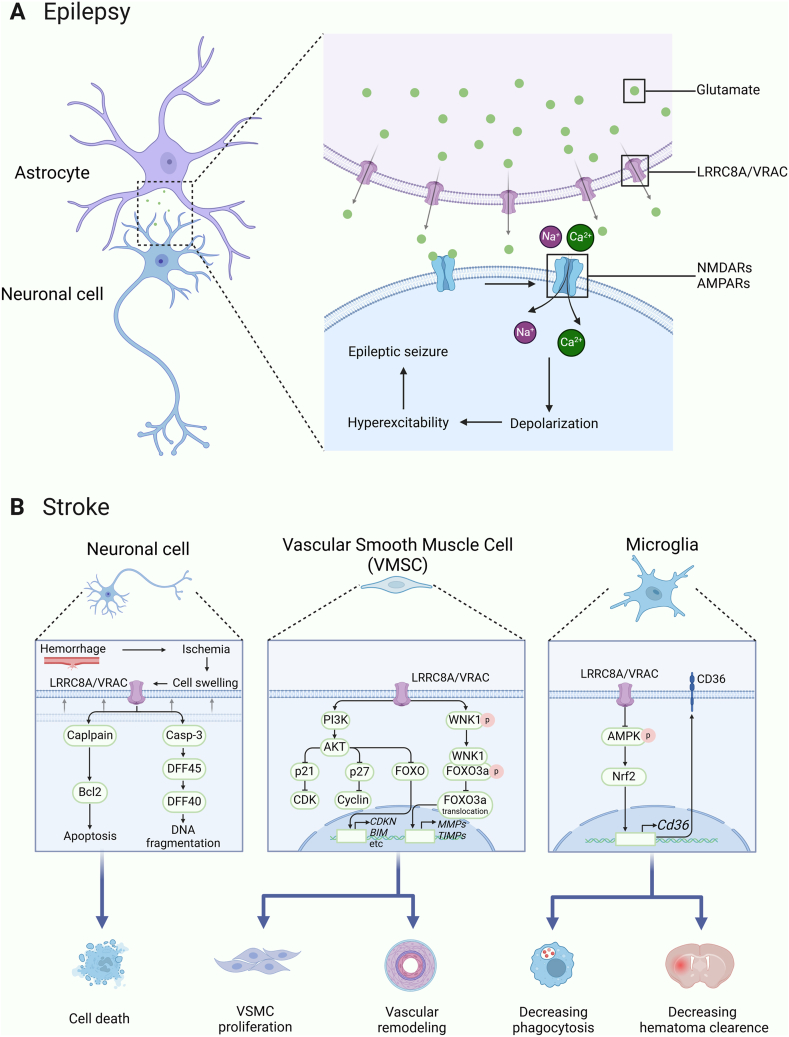
LRRC8A plays an important role in neurological diseases. **(A)** Astrocytes can release glutamate through LRRC8A/VRAC to increase neuronal excitability, thereby increasing the risk of epileptic seizures. **(B)** LRRC8A exacerbates stroke by promoting neuronal cell death, VSMC proliferation, and vascular remodeling; decreasing phagocytosis and hematoma clearance. NMDAR, N-methyl-d-aspartate receptor; AMPAR, α-amino-3-hydroxy-5-methyl-4-isoxazolepropionic acid receptor; VSMC, vascular smooth muscle cell.

Previous studies have reported that LRRC8A is associated with neurological disorders such as epilepsy and ischemic stroke. In this context, we elaborate on some confirmed mechanisms (Fig. 3).

### LRRC8A and epilepsy

Epilepsy is a neurological disorder characterized by recurrent seizures resulting from excessive neuronal excitation or synchronized discharge in the brain.^46^ The exact underlying causes of epilepsy remain incompletely understood, posing a barrier to the advancement of antiepileptic drug development. LRRC8A is predominantly up-regulated in astrocytes located around lesions in epilepsy patients.^47^ Astrocyte swelling triggers glutamate release through LRRC8A/VRACs, consequently increasing the excitability of neighboring neurons and serving as a crucial factor in seizure initiation.^48^^,^^49^ Neuronal excitability is highly sensitive to changes in extracellular osmotic pressure, and VRACs play a role in balancing ions inside and outside of neurons, thereby influencing neuronal excitability.^50^^,^^51^

Chloride ions, for example, move in and out of neurons primarily through VRACs, significantly affecting whether neurons trigger action potentials.^52^^,^^53^ Thus, if mutations in LRRC8A lead to dysfunction or abnormality in VRACs, this may alter neuronal excitability, potentially increasing the risk of epileptic seizures.

Furthermore, LRRC8A/VRACs in neuronal cells are activated under hypoosmotic conditions to regulate neuronal volume.^54^^,^^55^ Thus, mutations in LRRC8A could disrupt this volume regulation and further impact neuronal function and stability. VRACs are also involved in regulating the balance of intracellular and extracellular calcium ions,^56^ and abnormalities in calcium ion signal transduction have been shown to induce seizures in mice.^57^ In summary, mutations in LRRC8A may increase the risk of epileptic seizures by influencing astroglial cell function, neuronal excitability, and calcium ion signaling pathways.

### LRRC8A and ischemic stroke

Ischemic stroke is an acute neurological disorder that arises due to reduced or blocked cerebral blood flow, causing insufficient oxygen and nutrient supplies to brain tissue.^58^ This condition is typically triggered by vascular blockages such as thrombi or atherosclerosis.^59^ Following cerebral ischemia, water intrudes into neurons, leading to increased cellular volume, which can ultimately result in cell death.^60^ Studies have shown that blocking VRACs with DCPIB or conditionally knocking out LRRC8A in astroglial cells can alleviate ischemic brain injury and improve the prognosis for stroke patients.^49^^,^^61^ A plausible explanation is that lower LRRC8A levels reduce calpain and cleaved-caspase-3 activity in ischemic brain tissue,^62^ given that elevated calpain activity significantly contributes to neuronal death post-cerebral ischemia.^63^^,^^64^ In addition, smooth muscle cell proliferation is a major factor contributing to cerebral vascular remodeling and stroke.^65^ Lower expression of LRRC8A decreases AngII-induced cerebral vascular smooth muscle cell proliferation by inhibiting the PI3K-AKT pathway and the WNK1/FOXO3a/MMP pathway.^12^^,^^66^ Furthermore, the inhibition of LRRC8A can activate AMPK, induce the nuclear translocation of Nrf2, and increase the transcription of CD36.^67^ As a pattern recognition receptor, CD36 can enhance the phagocytic function of microglia/macrophages.^68^ These findings underscore the potential of LRRC8A as a therapeutic target for ischemic stroke. In summary, extensive research highlights the crucial role of LRRC8A, an essential component of VRACs, in central nervous system conditions such as epilepsy and stroke, suggesting it as a promising target for therapeutic interventions in neurological disorders.

Despite significant advances in our understanding of LRRC8A's role in neurological disorders, several substantial limitations persist in the current research. In epilepsy investigations, while increased LRRC8A expression has been documented in perilesional astrocytes with potential implications for neuronal excitability via glutamate release, several questions remain unresolved: whether this up-regulation represents a causative factor or a consequential response to epileptogenesis; the precise glutamate threshold required for seizure initiation; and the cell type-specific functions of LRRC8A throughout the central nervous system. In the context of ischemic stroke, although LRRC8A inhibition demonstrates neuroprotective potential, uncertainty remains regarding the optimal therapeutic window, the complex interplay between multiple LRRC8A-mediated signaling cascades, and the specificity of DCPIB as an LRRC8A inhibitor utilized in experimental paradigms. Additionally, our comprehension of how LRRC8A hetero-oligomerizes with auxiliary subunits (LRRC8B-E) to form functionally distinct channels remains inadequate. The field lacks comprehensive analyses of human clinical specimens and sufficient investigations into gene–environment interactions involving LRRC8A polymorphisms. Notably, research has predominantly focused on acute manifestations, neglecting the potential contribution of LRRC8A to disease chronicity and neurological recovery. These knowledge gaps necessitate the development of more selective LRRC8A modulatory compounds, enhanced clinical translational studies, and investigations of spatiotemporal-specific LRRC8A activity across different disease phases to thoroughly assess its therapeutic potential in neurological pathologies.

### LRRC8A in metabolic diseases

**Figure 4:**
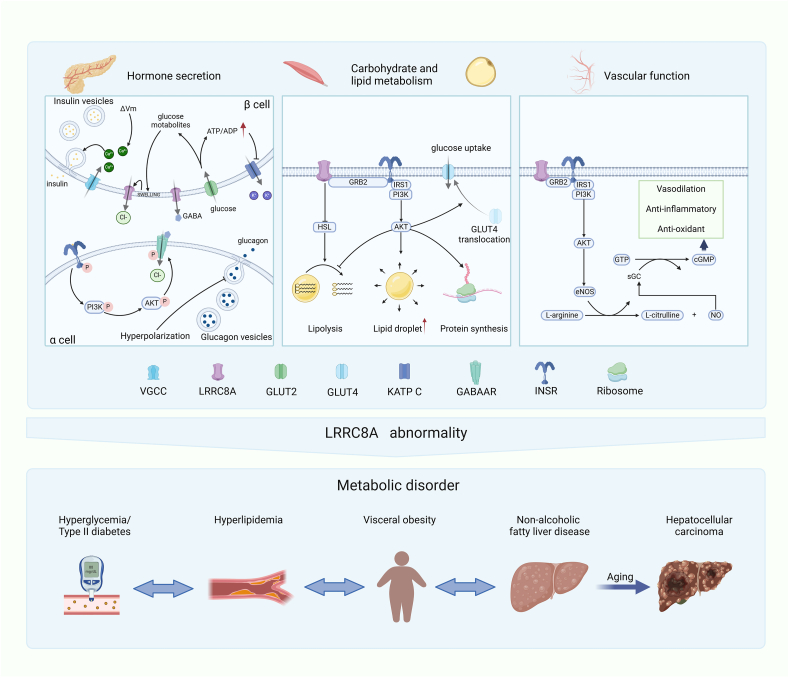
LRRC8A plays a vital role in maintaining normal hormone secretion in pancreatic islets, glucose and lipid metabolism, and vascular function. Therefore, dysfunction of LRRC8A can lead to metabolic disorders of glucose and lipids, potentially triggering type 2 diabetes and liver diseases.

LRRC8A abnormality is involved in various aspects of diabetes, including insulin resistance and impaired insulin secretion by pancreatic β-cells. Tissue-specific ablation of LRRC8A impairs insulin signaling in adipose, skeletal muscle, and endothelial cells and disrupts insulin secretion from pancreatic β-cells, ultimately compromising glucose homeostasis.^69^, ^70^, ^71^ This dysregulation possibly leads to metabolic disorders, increasing the risk of type 2 diabetes. Gamma-aminobutyric acid (GABA), secreted by pancreatic β-cells, plays a protective and regenerative role in β-cells.^72^ Meanwhile, GABA inhibits neighboring α-cells to prevent excessive glucagon release, which prevents further exacerbation of hyperglycemia.^73^^,^^74^ GABA also increases the number of β-cells in rodent and transplanted human islets,^75^, ^76^, ^77^ and improves glycemia in non-obese diabetic (NOD) mice.^78^ In pancreatic β-cells, cytosolic GABA is released via the LRRC8A/VRAC channel in a glucose concentration-independent pulsatile manner,^79^ highlighting the important regulatory role of LRRC8A/VRACs in β-cell insulin secretion. In the process of adipocyte hypertrophy, LRRC8A is activated by increased cell volume. Through interaction with GRB2-Cav1-IRS1-IR at its C-terminal leucine-rich repeat domain (LRRD), LRRC8A enhances insulin-PI3K-AKT2 signaling, thereby supporting insulin-mediated GLUT4 PM translocation, glucose influx, and lipogenesis.^10^ Thus, adipose tissue-specific knockout of LRRC8A (Adipo KO) under obese conditions could exacerbate insulin resistance and impair glucose uptake, potentially escalating liver disease from steatosis to HCC.^80^ Knockout of LRRC8A also inhibits insulin-mediated PI3K-AKT, ERK1/2, and mTOR signaling in skeletal muscle cells, thereby controlling skeletal muscle cell size, intracellular signaling, fat accumulation, and glucose metabolism, thereby influencing glucose uptake and utilization in muscle tissue.^81^ Additionally, LRRC8A regulates eNOS (endothelial nitric oxide synthase) signaling in endothelial cells through a GRB2-Cav1-eNOS complex, which is crucial for endothelial cell alignment to laminar shear flow.^82^ This regulatory mechanism influences vascular function and affects the progression of diabetes. In summary, the involvement of LRRC8A in cell volume regulation and insulin signaling makes it an integral part of various aspects of diabetes (Fig. 4).

### Role of LRRC8A in the immune system

LRRC8A plays a vital role in immune regulation, particularly in the development and function of T lymphocytes.^83^ Compared to that of other immune cells (splenic CD3^+^ T cells, B220^+^ B cells, DX5^+^ NK cells, CD14^+^ macrophages, and CD11c^+^ dendritic cells), the expression of LRRC8A on thymic cells is greater.^83^ Deletion of LRRC8A significantly impairs both the development and function of thymic T cells in mice.^84^, ^85^, ^86^ Recent studies have uncovered the dual role of LRRC8A in T cell development: it functions as an essential component of VRACs and a receptor that interacts with specific ligands expressed on thymic epithelial cells (TECs). These TEC-expressed ligands bind to the extracellular domain of LRRC8A on developing thymocytes, particularly during the crucial transition from double-negative (DN) to double-positive (DP) stages. This interaction triggers the LCK-ZAP-70-GAB2-PI3K signaling cascade, resulting in AKT phosphorylation—a process vital for thymocyte survival and proliferation.^87^ Additionally, LRRC8A is involved in regulating the cytokine production of T cells. Inhibition or genetic knockout of LRRC8A impairs T cell activation, affecting the production and secretion of key cytokines, such as TNFα and IFNγ.^85^ This may be due to impaired TCR (T cell receptor) signaling. During T-cell activation, antigen recognition by the TCR triggers a series of intracellular signaling events, leading to increased cellular metabolic activity and cell volume.^88^^,^^89^ In this process, LRRC8A controls the fine-tuning of the cell volume, maintaining an appropriate density of TCR signaling molecules and thereby ensuring effective TCR signal transmission. This is particularly significant under weak TCR signals, preventing the dilution of TCR molecules from cell expansion, thereby facilitating full T-cell activation.^86^ To sum up, LRRC8A is crucial for proper TCR signal transmission, a prerequisite for T cell activation, proliferation, and cytokine production. Without functional LRRC8A, T cells are compromised in producing cytokines that are critical for immune responses. Recent research has shown that the expression of LRRC8A in pancreatic adenocarcinoma is significantly associated with CD8^+^ T cells, neutrophils, and dendritic cell infiltration and influences the clinical prognosis of patients.^36^ These findings reveal the critical role of LRRC8A in the development, survival, and function of T cells.

In addition, LRRC8A/E VRACs are involved in the transport of immune signaling molecules such as cGAMP (2'3'-cyclic guanosine monophosphate-adenosine monophosphate),^90^ which induces a strong IFN response.^91^ Recent studies have indicated that extracellular ATP in the tumor microenvironment promotes cGAMP transfer, thus promoting STING signaling and IFN-β responses in mouse macrophages and fibroblasts. These effects diminish with chemical inhibition or reduced LRRC8A expression.^92^ Other studies have demonstrated that LRRC8A/VRACs may mediate the efflux of intracellular ATP.^93^^,^^94^ These findings highlight the key role of LRRC8A/VRAC in the cross-talk between extracellular ATP and cGAMP. LRRC8A also participates in the activation of NLRP3 inflammasome under hypo-osmotic stress, though it is not necessary for the danger-associated molecular pattern (DAMP)-induced NLRP3 activation.^95^^,^^96^ These findings suggest that LRRC8A is an important regulator of certain types of inflammasome activation.

**Figure 5:**
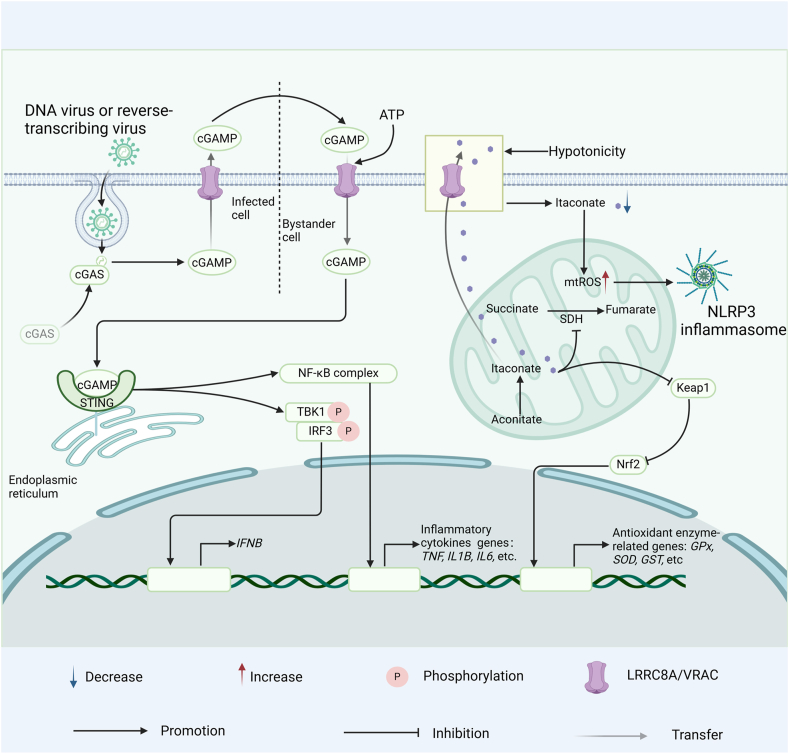
LRRC8A in the immune system. LRRC8A/VRAC activates the STING signaling pathway in bystander cells by transporting cGMP, thereby inducing antiviral immunity. LRRC8A also participates in the activation of the NLRP3 inflammasome under hypotonic stress. STING, stimulator of interferon genes; cGMP, cyclic guanosine monophosphate-adenosine monophosphate; NLRP3, NLR family pyrin domain containing 3.

Indeed, LRRC8A is also expressed on the surface of B cells, suggesting its potential role in regulating B cell function and immune responses, and maintaining B cell homeostasis.^97^ However, the specific role of LRRC8A in B cell function is not well defined, and further research is required to fully understand its impact on B cell function. In summary, LRRC8A contributes to immune regulation by supporting T cell development and function, modulating cell volume, facilitating the transport of immune signaling molecules, and participating in inflammasome activation (Fig. 5).

### Signaling pathways involving LRRC8A

**Figure 6:**
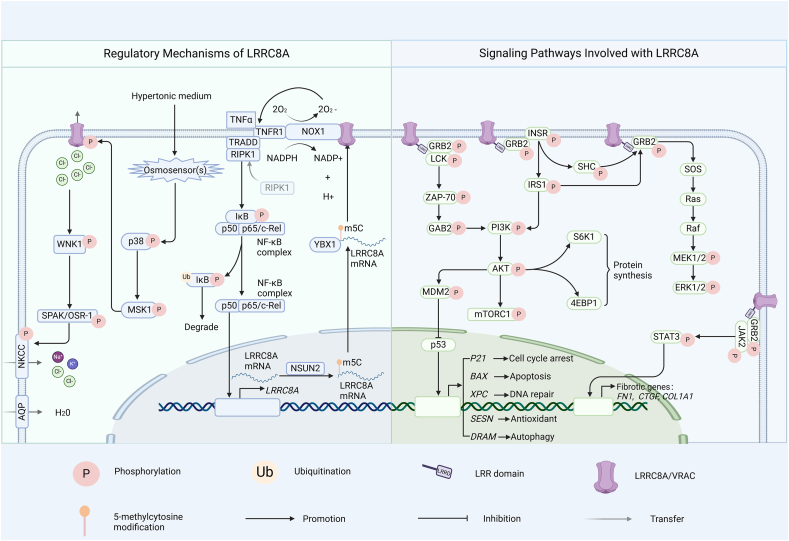
LRRC8A involved mechanisms. Regulatory mechanisms of LRRC8A: LRRC8A activates its own transcription by binding to NOX1 and activating the NF-κB signaling pathway. NSUN2 modifies LRRC8A mRNA with m5C, enhancing its stability and thus increasing LRRC8A expression. MSK1 phosphorylates and activates LRRC8A, aiding cell volume recovery and survival under hypertonic conditions. Signaling pathways involving LRRC8A: LRRC8A activates the PI3K-AKT signaling pathway, insulin signaling pathway, MAPK signaling pathway, and JAK-STAT signaling pathway through its LRR domain by interacting with GRB2.

LRRC8A participates in various signaling pathways through its interaction with different proteins and complexes. Some key signaling pathways involving LRRC8A are as follows (Fig. 6).

### cGAS-STING pathway

The STING signaling pathway is a critical component of cellular innate immune responses, capable of sensing cytosolic DNA and activating interferon production to counteract viral infections and tumor development.^98^, ^99^, ^100^, ^101^ cGAMP, a signaling molecule synthesized by cyclic GMP-AMP synthase (cGAS) upon recognition of cytoplasmic DNA, is crucially translocated across the cell membrane by LRRC8A/VRAC.^90^^,^^102^^,^^103^ When an organism is infected, cGAMP is transported from infected cells to uninfected surrounding cells via LRRC8A/VRAC. This transport process is crucial for activating the STING signal in surrounding cells.^90^ Upon binding with cGAMP, STING can activate the downstream TANK binding kinase 1 interferon regulatory factor 3 (TBK1-IRF3) signaling pathway, inducing the expression of IFN, thus initiating the host antiviral immune response.^91^ In addition to hypo-osmotic conditions, inflammatory factors such as TNF and IL-1 can also activate VRACs, thereby promoting cGAMP transport and STING signal activation.^90^ These studies suggest that LRRC8A/VRAC also plays a significant role in inflammatory responses and immune activation. In VRAC knockout mouse models, HSV-1 infection results in reduced IFN responses and increased viral loads in these models, further confirming the critical role of LRRC8A/VRAC in antiviral immunity.^90^ The cGAMP transport and STING signal activation, regulated by LRRC8A/VRAC, not only play an essential role in antiviral immunity but also provide new ideas for cancer immunotherapy. By affecting immune responses in the tumor microenvironment, LRRC8A/VRAC may play a role in tumor immune surveillance and immunotherapy. In summary, LRRC8A, through its key role in VRAC, facilitates the transport of cGAMP, thereby activating the STING signaling pathway, which has a significant impact on antiviral immunity and antitumor immunity. The discovery of this molecular mechanism not only provides a new perspective for understanding immune regulation in the body but also offers potential targets for vaccine development and immunotherapy, with significant scientific importance and application prospects.

### PI3K-AKT pathway

The PI3K/AKT signaling pathway serves as a crucial regulator of various physiological and pathological cellular processes, including cell proliferation, survival, and invasion.^104^, ^105^, ^106^ This pathway consists of two main components: PI3K, which phosphorylates PIP2 to produce PIP3, and AKT, which is activated upon binding to PIP3 through its pleckstrin homology (PH) domain.^107^^,^^108^ Once activated, AKT phosphorylates a diverse array of substrates, particularly targeting mechanistic target of rapamycin complex 1 (mTORC1) effectors such as eukaryotic translation initiation factor 4E-binding protein (4EBP1) and ribosomal protein S6 kinase polypeptide 1 (S6K1), thereby promoting protein synthesis and cellular proliferation.^109^

LRRC8A facilitates an interaction with the SH3 domains of growth factor receptor-bound protein (GRB2) and lymphocyte-specific protein tyrosine kinase (LCK) through the proline-rich region in its first intracellular structural domain. This interaction may occur directly or indirectly via the involvement of growth factor receptor-bound protein 2-associated binding protein (GAB2) with the GRB2-LCK complex. Such interactions lead to the phosphorylation of LCK, along with its substrate zeta-chain-associated protein kinase 70 (ZAP-70) and the target GAB2 at residue Y452.^110^ Following this, PI3K is recruited to GAB2, where it undergoes phosphorylation by LCK, subsequently triggering the activation of AKT through its phosphorylation.^111^^,^^112^ Notably, LRRC8A-mediated AKT activation can be inhibited by sarcoma viral oncogene homolog (SRC), spleen tyrosine kinase (SYK)/ZAP-70, and PI3K inhibitors.^83^ These findings indicate that various members of the SYK and SRC families of kinases may participate in LRRC8A-mediated AKT activation; however, the precise mechanisms warrant further investigation.

### JAK2-STAT3 pathway

The Janus kinase-signal transducer and activator of transcription (JAK-STAT) pathway is involved in various cellular processes, including cell growth, differentiation, immune responses, and the cellular stress response.^113^ Activation begins when extracellular signaling molecules, such as cytokines or growth factors, bind to cell surface receptors, leading to the activation of associated JAK kinases. These kinases, in turn, phosphorylate and activate STAT proteins, which subsequently dimerize and translocate to the nucleus, functioning as transcription factors that modulate the expression of target genes.^114^ Dysregulated JAK-STAT pathway activation has been implicated in a range of pathologies, including cancer, autoimmune diseases, and chronic infections.^115^, ^116^, ^117^

LRRC8A interacts with GRB2 through its C-terminal LRRD,^118^ an adaptor protein that associates with and is essential for tyrosine-phosphorylated JAK2.^119^ This interaction facilitates the activation of the JAK2-STAT3 signaling pathway and the induction of a myofibroblast phenotype under TGF-β1 stimulation. Notably, silencing LRRC8A has been found to mitigate this effect.^118^

### The p53 signaling pathway

The p53 signaling pathway plays key roles in maintaining genomic stability and preventing the development of cancer.^120^ The core effector molecule p53 is activated in response to various cellular stresses, such as DNA damage, hypoxia, and oncogene activation. Once activated, p53 regulates the expression of genes involved in cell cycle arrest, apoptosis, senescence, and DNA repair.^121^, ^122^, ^123^ LRRC8A participates in modulating the p53 signaling pathway in gastric cancer, highlighting its role in cell cycle regulation and apoptosis.^30^ While the specific interaction details between LRRC8A and the p53 pathway may vary depending on the cell type and environment, we outline the likely mechanisms here based on known interactions and effects.

First, LRRC8A affects gene expression, controlling cell cycle arrest and apoptosis by influencing the expression of p53 and its downstream target genes. For instance, silencing LRRC8A in gastric cancer cells increases the expression of p53 and several related genes, such as p21 (a CDK inhibitor induced by p53), Bcl-2 (an anti-apoptotic protein), and FAS (a death receptor), which together inhibit cell proliferation and promote apoptosis. These findings suggest that LRRC8A may facilitate cancer cell survival by modulating the p53 pathway, influencing the stability or transcriptional activity of p53 and related genes, thereby indirectly regulating cell proliferation and apoptosis.^30^ Additionally, the influence of LRRC8A on the p53 pathway might involve interactions with pathways converging at p53 or regulated by p53. For example, AKT can phosphorylate MDM2, facilitating its nuclear translocation.^124^ MDM2 acts as a negative regulator of p53, promoting its degradation and inhibiting its activity.^125^, ^126^, ^127^ Thus, LRRC8A might influence the p53 pathway through the AKT pathway. This points to a complex interaction network in which LRRC8A might indirectly affect p53 activity by engaging in other signaling pathways. In summary, LRRC8A influences cell proliferation and apoptosis by regulating the expression of p53 and its target genes. This regulation may occur directly through the modulation of gene expression or indirectly via interactions with pathways that control p53 activity. Further research is needed to elucidate the precise mechanisms by which LRRC8A affects the p53 pathway, especially given that its regulatory effects may vary across different cellular contexts and disease states.

Additionally, LRRC8A can also regulate skeletal muscle size by affecting the insulin-PI3K-AKT signaling pathway.^81^ Interactions between LRRC8A and both the p53 and PI3K-AKT pathways might also affect other signaling pathways, such as the MAPK pathway, which is essential for maintaining genomic stability and preventing cancer development.^128^^,^^129^ These studies suggest that LRRC8A may play various roles in regulating intracellular signaling cascades, potentially impacting several cellular processes involved in immune regulation, cancer onset and progression.

### Regulatory mechanisms of LRRC8A

Current research indicates that the expression of LRRC8A is primarily influenced by post-translational modifications and cellular signaling pathways (Fig. 6).

### Self-regulated mechanism of LRRC8A

Recent research has discovered that LRRC8A plays a vital role in regulating its own expression, a process involving interaction with Nox1 (NADPH oxidase 1) and activation of the NF-κB signaling pathway. In vascular smooth muscle cells, LRRC8A interacts with Nox1, a key enzyme involved in the production of reactive oxygen species, promoting the production of superoxide radicals (O2•-), which in turn facilitates tumor necrosis factor receptor 1 (TNFR1) endocytosis and activates the downstream NF-κB signaling pathway.^130^, ^131^, ^132^ The NF-κB signaling pathway is an important intracellular signaling pathway involved in regulating various cellular responses, including the immune response, cell proliferation, and apoptosis.^133^^,^^134^ Through activating the NF-κB pathway, LRRC8A enhances the transcriptional activity of its own gene. This self-regulatory mechanism allows LRRC8A to respond rapidly when cells are stimulated by tumor necrosis factor alpha (TNF-α), increasing its protein expression levels and thereby strengthening the ability of cells to respond to TNF-α signals. This mechanism plays a significant role in the development of tumors such as colorectal cancer.^33^

### The m5C modification-mediated mRNA regulation of LRRC8A

The m5C modification is a post-transcriptional modification that involves the addition of a methyl group to the 5th carbon position of cytosine in RNA (m5C), which can influence various aspects of mRNA metabolism, including its stability, localization, and translation efficiency.^135^^,^^136^ The m5C modification is catalyzed by the m5C methyltransferase family, such as NSUN2, which is responsible for the m5C modification of specific RNA substrates.^137^^,^^138^ In cervical cancer cells, NSUN2 mediates the m5C modification of LRRC8A mRNA, allowing m5C-modified LRRC8A mRNA to bind with the RNA-binding protein YBX1 (Y-Box Binding Protein 1),^35^ thus increasing the stability of the mRNA. This binding enhances the stability of the LRRC8A mRNA, increasing its translation and, consequently, LRRC8A protein levels. In summary, the m5C modification promotes the binding of LRRC8A mRNA to YBX1 through methylation mediated by NSUN2, which enhances the stability of LRRC8A mRNA and promotes the expression of LRRC8A, thereby playing its biological function in tumor cells.

### The phosphorylation of LRRC8A

The p38 mitogen-activated protein kinase (MAPK) pathway plays a crucial role in regulating the activity of the LRRC8A channel under hypertonic conditions.^139^^,^^140^ The channel activity of VRAC, which contains LRRC8A, is affected both by the p38 inhibitor SB203580 and in p38α knockout cells, indicating that the p38 signaling pathway is involved in regulating the opening mechanism of VRAC channels.^139^ Mitogen and stress-activated kinase 1 (MSK1) is a downstream kinase of p38.^141^^,^^142^ Hypertonic stress triggers MSK1 phosphorylation, and the inhibition of MSK1 prevents LRRC8A phosphorylation under these conditions, highlighting the importance of MSK1-mediated phosphorylation for LRRC8A activity in hypertonic environments.^139^ Specifically, several presumed MSK1 phosphorylation sites are contained in the intracellular loop of LRRC8A. Mutation of serine 217 to alanine almost completely abolishes the phosphorylation in this region induced by MSK1, suggesting that S217 is crucial for MSK1-mediated phosphorylation and activation of LRRC8A.^139^ These findings underscore the role of the p38 MAPK pathway and MSK1 in regulating LRRC8A, supporting the recovery of cellular volume and survival under hypertonic stress.

### LRRC8A as a therapeutic target

While no drugs have been developed specifically to target LRRC8A, increasing research underscores its therapeutic potential, particularly through the modulation of VRAC, in which LRRC8A is a critical component. VRAC is integral to cellular osmotic regulation and plays a pivotal role in cancer biology, neurological disorders, and immune response modulation. Consequently, inhibiting or modulating LRRC8A and VRAC could present promising new avenues for the treatment of these diseases.

#### Potential applications of LRRC8A in disease therapy

##### Chemotherapy sensitization

As a core component of VRAC, LRRC8A plays a crucial role in modulating the efficacy of chemotherapeutic agents in cancer treatment. Research has shown that LRRC8A impacts tumor cell drug resistance by influencing the cellular responsiveness to chemotherapy.^28^ The VRAC channel formed by LRRC8A also facilitates the transport of certain small-molecule chemotherapeutics, such as cisplatin, which is essential for optimal therapeutic effects.^143^ Thus, targeting LRRC8A function might be a promising strategy to improve drug uptake, reduce resistance, and increase the efficacy of chemotherapeutic regimens in resistant cancers, offering a new avenue for improving treatment efficacy.

Immunoregulatory role of LRRC8A: LRRC8A plays a crucial role in the activation, differentiation, and functional regulation of immune cells by facilitating volume and ion homeostasis. Its activity is crucial in mediating T cell activation and proliferation, where LRRC8A supports optimal signaling for effective immune responses. Additionally, LRRC8A enhances pro-inflammatory signaling pathways within macrophages, influences their migration to inflammatory sites, and modulates their cytokine production, reinforcing immune responses in inflammatory contexts.^85^^,^^144^ These findings position LRRC8A as a promising target for modulating immune function. Inhibiting LRRC8A activity may mitigate abnormal immune cell activation and cytokine release, thereby alleviating symptoms associated with autoimmune diseases. Furthermore, the immunosuppressive tumor microenvironment is a key factor driving immune tolerance and limiting the efficacy of immunotherapy. By modulating immune cell functions, LRRC8A enhances anti-tumor immune responses, providing a novel avenue to improve immunotherapeutic strategies.

Given the widespread expression of LRRC8A across tissues and the diverse functions of LRRC8A-formed VRAC channels in different cell types, the straightforward activation or inhibition of LRRC8A may not be sufficient for effective disease treatment. However, recent advancements in gene-editing technologies, such as CRISPR-Cas9, now allow for cell type-specific regulation of target genes.^145^ This emerging strategy for targeted immune modulation may facilitate the precise development of drugs that specifically target LRRC8A in selected cells.

### Known VRAC inhibitors and modulators

DCPIB: DCPIB is a selective VRAC inhibitor that effectively blocks VRAC activation, inhibiting the efflux of chloride ions and organic solutes essential for cell volume regulation.^146^ Given the important role of VRACs in tumor cell apoptosis, DCPIB is frequently employed in studies examining the response of cancer cells to chemotherapeutic agents. For instance, *in vitro* investigations have demonstrated that the inhibition of VRAC can enhance the efficacy of specific chemotherapy drugs, such as paclitaxel, in ovarian cancer cells.^147^

Tamoxifen: Tamoxifen is a non-specific VRAC inhibitor primarily utilized as an anti-estrogen medication in the treatment of breast cancer.^148^ However, it has also been identified as a VRAC inhibitor.^149^ Studies suggest that Tamoxifen can induce cell cycle arrest in cervical cancer cells by modulating VRAC channel activity.^150^

Non-steroidal anti-inflammatory drugs (NSAIDs): Certain NSAIDs, including FFA (flufenamic acid) and MFA (mefenamic acid), have been shown to inhibit VRAC activity, thereby diminishing the formation of the NLRP3 inflammasome.^96^^,^^151^^,^^152^

## Future research directions

### Structure and function

The elucidation of the three-dimensional structure‒function relationship of LRRC8A necessitates further comprehensive investigation. Although recent advances in cryo-electron microscopy have illuminated partial structural characteristics of LRRC8A/LRRC8C heterohexamers, our current understanding of the molecular mechanisms through which various LRRC8 subunit combinations modulate channel functionality remains inadequate.^153^ Addressing this knowledge deficit requires high-resolution structural analyses via cryo-electron microscopy and X-ray crystallography to delineate the complete architecture of diverse LRRC8 subunit combinations, with a particular emphasis on the conformational dynamics in heterohexamers comprising LRRC8A with LRRC8B-E under various physiological conditions. Furthermore, elucidating the role of LRRC8A within protein–protein interaction networks is essential, especially for examining how these interactions influence signal transduction pathways and channel gating mechanisms. The development of highly specific small-molecule modulators targeting discrete LRRC8A domains would provide valuable investigative tools for VRAC channel research while simultaneously offering potential therapeutic candidates for associated pathological conditions. Through such systematic methodological approaches, we may comprehensively elucidate the pivotal functions of LRRC8A in cellular physiological and pathological processes.

### LRRC8A in cancer

LRRC8A demonstrates differential expression patterns across diverse tumor types, exhibiting heterogeneous and occasionally antagonistic functions in various malignancies. This complexity necessitates sophisticated research methodologies to delineate its precise role in neoplastic biology. Systematic profiling of LRRC8A expression signatures and mutational landscapes across tumor subtypes, integrated with clinical outcome data for predictive modeling, would facilitate personalized therapeutic interventions. Furthermore, investigations of novel LRRC8A-associated signaling networks, specifically those implicated in metabolic reprogramming, cell cycle dysregulation, and immune evasion strategies, will illuminate its multifaceted contributions to tumor progression. These investigations may ultimately inform the development of LRRC8A-targeted therapeutic modalities, including selective inhibitors for tumors with LRRC8A overexpression or adjuvant approaches that exploit LRRC8A-mediated pathways to potentiate conventional chemotherapeutic efficacy. These research directions are poised to investigate the potential role of LRRC8A in tumor heterogeneity, which may contribute to our understanding of precision oncology frameworks.

### LRRC8A in neurological disorders

LRRC8A serves as a critical mediator in neural development and homeostasis, with its dysregulation implicated in diverse neurological pathologies. To elucidate its comprehensive neurobiological significance, future investigations should address multiple hierarchical dimensions. First, conditional LRRC8A knockout mouse models would enable cell-specific analyses across neural populations (neurons, astrocytes, and oligodendrocytes), revealing distinct roles of LRRC8A in different cell types. Second, mechanistic studies should examine the modulatory effects of LRRC8A on synaptic plasticity, neurotransmitter dynamics, and neuronal excitability. Particularly, how its volume-regulatory functions influence signal transduction pathways, thereby illuminating its mechanistic contributions to neurophysiological processes, is unclear. Furthermore, investigation of the influence of LRRC8A on blood‒brain barrier integrity and its paradoxical roles in neuroprotection versus neurodegeneration following ischemic or traumatic insults could clarify its complex functions in neuropathological contexts. Ultimately, these mechanistic insights could inform the development of therapeutic interventions targeting LRRC8A, potentially through the modulation of volume-regulated anion channel activity to attenuate excitotoxicity or neuroinflammatory cascades, thereby opening novel avenues for neurological disease management.

### LRRC8A in metabolic diseases

The emerging roles of LRRC8A in pancreatic β-cell function, adipose tissue differentiation, and hepatic glucose homeostasis have been documented, yet its precise regulatory mechanisms remain incompletely characterized. A comprehensive investigation of the metabolic functions of LRRC8A will advance the understanding of and therapeutic approaches to metabolic disorders. Delineating the signaling cascades through which LRRC8A modulates insulin secretion and β-cell viability could reveal novel targets for early intervention in type 2 diabetes pathogenesis. Building on this foundation, an examination of the molecular mechanisms of LRRC8A in adipocyte differentiation, lipid metabolism, and insulin sensitivity could provide complementary insights and potentially identify therapeutic strategies for addressing the global obesity epidemic. Similarly, important is the investigation of the contribution of LRRC8A to hepatic metabolic dysregulation, particularly in non-alcoholic fatty liver disease, as this will enhance our understanding of its regulatory effects on lipid homeostasis, oxidative stress, and inflammatory pathways. Extending these investigations to skeletal muscle, characterizing the involvement of LRRC8A in insulin resistance, including its interactions with glucose transporters and insulin signaling components, may uncover previously unrecognized mechanisms underlying systemic metabolic dysfunction. Ultimately, developing LRRC8A-targeted metabolic modulators represents a promising strategy for treating metabolic syndrome and related disorders. Through these systems-level research approaches, we may better understand the potential role of LRRC8A in energy metabolism and metabolic diseases, which may contribute to the development of new frameworks for precision metabolic medicine.

### Translational medicine of LRRC8A

Translating fundamental research findings into clinical applications represents a critical direction in LRRC8A research. Studies have demonstrated that LRRC8A can be secreted extracellularly via exosomes. Elucidating the correlation between LRRC8A expression levels and tumorigenesis while developing non-invasive methods to detect LRRC8A expression and activity, would enable its utilization as a biomarker for early tumor detection and prognostic evaluation. This approach could significantly enhance early disease identification and therapeutic intervention strategies. Moreover, the development of LRRC8A-specific drug delivery systems that exploit differential VRAC activity across tissue types offers potential for targeted therapeutics, enhancing efficacy while minimizing systemic adverse effects. For example, VRAC activity differs significantly between cancer and normal cells.^154^ By identifying specific ligands for LRRC8A and conjugating chemotherapy drugs with these LRRC8A-specific ligands, the drugs can be primarily released in cancer cells with high VRAC activity but have minimal impact on normal cells with low VRAC activity, thereby reducing the side effects of chemotherapy. Exploring the interactions between LRRC8A and commonly used clinical drugs, optimizing existing treatment regimens, and improving the efficacy and safety of clinical medications are equally valuable research directions. Through this comprehensive exploration from diagnosis to treatment, LRRC8A research will likely achieve effective translation from the laboratory to clinical practice, providing new solutions for precision medicine across various diseases.

## Conclusion

LRRC8A research is advancing rapidly, revealing multifunctional roles beyond volume regulation that offer significant insights into disease mechanisms and therapeutic development. Future investigations should prioritize elucidating structure‒function relationships, tissue-specific regulation, and network interactions within pathological contexts. Particularly valuable will be the integration of LRRC8A within disease-specific signaling pathways, contextualizing its functions in broader physiological frameworks.

## CRediT authorship contribution statement

**Longjun Yang:** Writing – original draft. **Qiang Ding:** Visualization. **Xiaoyu Ji:** Visualization. **Panpan Lu:** Writing – review & editing. **Mei Liu:** Supervision.

## Funding

This work was supported by the Key Research and Development Projects of Hubei Province, China (No. SCZ202111), the 10.13039/501100001809National Natural Science Foundation of China (No. 82203793), and the Postdoctoral Science Foundation of China (No. 2024M761032).

## Conflict of interests

The authors declare that they have no competing interests.
